# Is Teledentistry a Method for Optimizing Dental Practice, Even in the Post-Pandemic Period? An Integrative Review

**DOI:** 10.3390/ijerph19137609

**Published:** 2022-06-22

**Authors:** Andreea Kui, Codruța Popescu, Anca Labuneț, Oana Almășan, Adrian Petruțiu, Mariana Păcurar, Smaranda Buduru

**Affiliations:** 1 Prosthetic Dentistry and Dental Materials Department, Iuliu Hatieganu University of Medicine and Pharmacy, 32 Clinicilor Street, 400006 Cluj-Napoca, Romania; andreeakui@gmail.com (A.K.); labunet@yahoo.com (A.L.); oana.almasan@umfcluj.ro (O.A.); smarandabudurudana@gmail.com (S.B.); 2 Department of Abilities Human Sciences, Iuliu Hatieganu University of Medicine and Pharmacy, 400012 Cluj-Napoca, Romania; 3 Department of Periodontology, Iuliu Hatieganu University of Medicine and Pharmacy, 400012 Cluj-Napoca, Romania; stefan.petrutiu@umfcluj.ro; 4 Orthodontic Department, George Emil Palade University of Medicine, Pharmacy, Science and Technology, 38 Gheorghe Marinescu Street, 540142 Targu Mures, Romania; mariana.pacurar@umfst.ro

**Keywords:** teledentistry, COVID-19 pandemic, post-pandemic period, dental care

## Abstract

Background: For the past two and half years, dentists all across the world, along with their patients, have faced numerous challenges. In this context, the aim of this integrative review was to assess if dentists’ and patients’ attitudes regarding teledentistry (TD) have changed since the COVID-19 outbreak, and if the use of TD will continue to rise, even in the post-pandemic period; (2) Methods: A literature search was performed between August 2021 and January 2022. The PubMed, Scopus, and Science Direct databases were searched for articles published between 2012 and 2022 using a combination of the following Mesh terms: “COVID-19”, “pandemic”, “oral telemedicine”, “teledentistry”; (3) Results: Among the 52 included papers, nine papers were published between 2011 and 2019, and 43 articles were published after 2020 (12 were published in 2020, 29 papers were published in 2021, and two in 2022). Among the articles published before 2020, seven out of nine included papers were reviews, and two were original research. Among the 43 papers published after 2020, 18 were reviews and 25 original research. (4) Conclusions: Based on the results of this integrative review, there is clear evidence that the interest in teledentistry and teleassistance in the dental field has increased rapidly, especially in the context of the COVID-19 pandemic. Therefore, while dental practitioners should be encouraged to keep themselves updated about new technologies, patients should also be constantly informed about their options for receiving special oral health care.

## 1. Introduction

Since the outbreak of the COVID-19 pandemic, health services, including dental practices, have been severely disrupted. Therefore, dentists and patients have had to overcome different challenges for the last two years. The difficulties emerged from the high risk of droplet and aerosol exposure, as SARS-CoV-2 is transmitted mostly by contact with infected droplets, either directly or indirectly, and especially in the presence of highly concentrated aerosols in closed environments [1,2]. In dental practice, aerosol generating procedures (AGPs) represent a health concern, as aerosols during clinical procedures can become contaminated with microorganisms, which can cause respiratory health effects or spread infections bidirectionally, among dental professionals and patients [2,3]. In addition, the overall amount of particulate matter (PM) produced during dental procedures is influenced by a variety of factors, such as the ventilation, type of procedure, and the use of standard saliva ejectors [2]. Under these circumstances, the conventional protective measures were no longer effective in the case of SARS-CoV-2 virus presence [4,5]. When evaluating the contamination produced after cavity preparation using a two-hole and four-hole handpiece, Ahmed et Jouhar (2021) found that the mean amount of aerosol and splatter produced by both handpieces was not statistically significant; however, the amount of aerosol and splatter produced at a distance of 12, 24, and 36 inches immediately after cavity preparation and 30 min after cavity preparation was statistically significant, regardless of the type of handpiece used [6]. While current guidelines suggest minimizing the use of airborne spreading devices (in order to reduce the production of PM particles), some studies have shown that avoiding natural ventilation during the performance of dental activities and using low-volume suction might considerably reduce the total amount of PM particles [2,7]. In addition, special precautions against aerosol transmission should be taken, and the use of aspirating systems with HEPA filters, which evacuate air and dissipate in the atmosphere, has been advocated [8].

Various countries globally have implemented stringent measures and initiatives to reduce intimate contact between people, in order to prevent the virus from spreading and to lower the risk of infection. Dental health care personnel (DHCP) and dental education institutions faced immediate issues, as a result of such actions [9,10]. All over the world, medical universities and dental schools had to adapt to new teaching conditions; coping with closures, social distancing, and remote continuing learning activities [11,12]. Onsite lectures and practical activities were changed to online lectures and e-learning technologies [12]. While those methods represented a common strategy, the ways in which these have been used were various and included live lectures or lecture streaming, comprising videos and links to several online apps, in order to simulate different clinical procedures and to create online assessments [11].

As a result, the current health crisis has presented new challenges to dentists, motivating new priorities, such as ensuring patient safety, regulating and decreasing contamination hazards, and increasing treatment efficiency to boost productivity and profitability. Therefore, new methods, tools, and a greater usage of digital health have emerged. In many ways, digital technology, such as intraoral scanners and in-office computer aided design/computer aided manufacturing (CAD/CAM) systems are the ideal instruments for dealing with these new issues. Dentists are increasingly embracing digital technologies, which may produce exact and exceptional results if practitioners adhere to current standards and best practices [13,14].

According to the World Health Organization (WHO), telemedicine is defined as “The delivery of health-care services, where distance is a critical factor, by all health-care professionals using information and communications technologies for the exchange of valid information for treatment and prevention of disease and injuries, research and evaluation, and the continuing education of health-care workers, with the aim of advancing the health of individuals and communities” [15,16]. Based on this definition, oral telemedicine or teledentistry (TD) can be described as the use of telemedicine in the field of oral health, particularly dentistry [10].

To the best of our knowledge, there is little information available regarding dentist’s expectations and attitude towards TD, especially in the context of COVID-19 pandemic.

By performing this integrative review, we aimed to investigate whether dentists’ attitudes and perspectives toward teledentistry have changed compared to the pre-pandemic period and if teledentistry might become a common method for optimizing dental practice in the future. In addition, we assessed patients’ perspectives regarding the use of teleassistance in dentistry.

## 2. Materials and Methods

This study used a five-step integrative review process that included (1) problem identification, (2) literature search, (3) data evaluation, (4) data analysis, and (5) presentation and interpretation of the findings [17].

### 2.1. Information Sources and Search Strategy

A literature search was performed between August 2021 and January 2022. PubMed, Scopus, and Science Direct databases were searched using a combination of the following Mesh terms: “COVID-19”, “pandemic”, “oral telemedicine”, “teledentistry” (Table 1). All references were imported and organized in the bibliographic software Mendeley^®^ (Mendeley Software, London, UK software company).

### 2.2. Selection of Articles

Articles published between 2012 and 2022 were included. Clinical trials, randomized controlled trials, reviews, and systematic reviews published in English language were considered to be eligible.

The exclusion criteria were, as follows: no full-text available, editorials, short communication, letter to editors, articles written in other language than English and studies outside of area of investigation.

### 2.3. Data Collection

**Figure 1:**
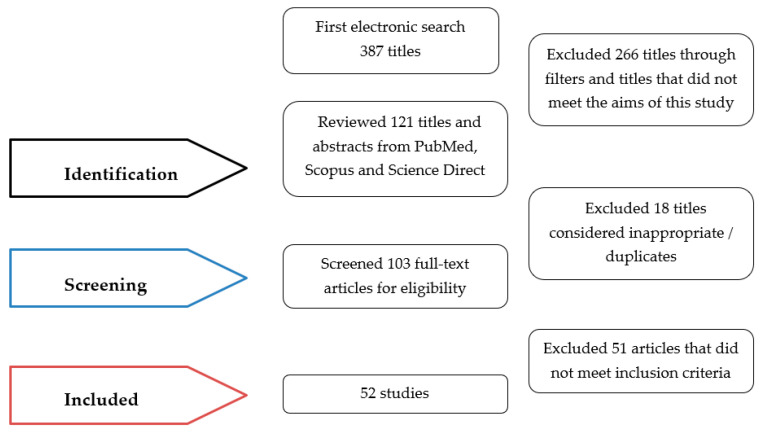
PRISMA flowchart.

All the citations that were imported into the bibliographic software Mendeley were double-checked, and duplicates were eliminated. The evaluations were carried out independently by two reviewers (A.K. and A.L.). Excel spreadsheets (Microsoft Office 2019^®^, MS, Redmond, WA, USA) were used to assess each publication. The following information was gathered using a standardized form: (1) authors’ names and publication year; (2) study design; (3) study goal; (4) methods; (5) main findings; and (6) conclusions. Afterwards, both investigators (A.K. and A.L.) compared their assessments and confirmed the data on the basis of the compiled spreadsheets. Both researchers compared their findings and verified the information. In case of any disagreement about the study data, the two researchers discussed, and a third researcher resolved, the issue (C.P.). The PRISMA statement rules were followed during the literature selection procedure [17] (Figure 1).

## 3. Results

### 3.1. Literature Research

A total of 387 papers were found using the search method. After eliminating 266 titles, 121 titles were reviewed. After excluding 18 titles (considered inappropriate or duplications) 103 articles were screened for eligibility. The authors individually screened the abstracts, in order to identify the papers that were relevant to the aims of the research and in accordance with the inclusion criteria.

After the full text reading of the remained studies, 51 publications were eliminated because they did not meet the inclusion criteria or did not match the outcomes of this paper. As a result, a total of 52 publications were included in this review.

### 3.2. Description of the Studies and Analysis

Nine papers were published between 2011–2019, 12 were published in 2020, 29 papers were published in 2021 and 2 in 2022.

Regarding the types of study, among the included 52 papers, 27 were original articles (5 cross-sectional studies, 2 case-control studies, 5 observational researches, 9 surveys, 4 prospective and retrospective studies, 2 case-reports), and 25 were reviews.

## 4. Discussions

As the COVID-19 pandemic has renewed attention regarding aerosol-generating procedures (AGPs), many publications since the pandemic emerged have focused on the essential response measures, by providing some management protocols for dentists and dental professionals. Particularly in dental practice, there are various procedures, such as close communication with patients, exposure to body fluids, use of equipment of aerosol generating tools, and airborne spread of saliva and other biological fluids [18]. Organizing dental activities and procedures, while protecting dental staff and patients from cross-infection, concerned dental practitioners and researchers. Measures such as managing patients, limiting the use of equipment considered to be high source of aerosol contamination (ultrasonic and sonic scalers, air polishing devices, high speed hand pieces), and establishing strict protocols, in order to prevent cross-infection in non-clinical and clinical areas in a dental office, proved to be effective when implemented [19]. In addition, telemedicine and teledentistry allowed promoting remote consultations and follow-ups without the physical presence in a medical or dental office. Using teledentistry (TD) allowed, not only replacing some of the onsite visits to dental offices, but also creating practitioner-to-practitioner connections by exchanging medical records, examination data, and therapeutic information [19,20,21].

### 4.1. The Use of Teledentistry before the COVID-19 Pandemic

Among the articles published before 2020, seven out of the nine included papers were reviews, and two were original research. In their study published in 2013, Kopycka-Kedzierawski and Billings conducted a comparative study investigating the effectiveness of teledentistry screening of early childhood caries (ECC) versus the traditional clinical examination screening method. Three hundred and forty-three children (age between 12–60 months) were included in the research and were divided into study groups. Based on a statistical analysis of the data, the authors concluded that oral screening of preschool children was comparable to classic visual oral examination [22]. In the research published by Kohara et al. (2018), the authors investigated two models of smartphones (iPhone and Nexus) and a conventional camera for detecting carious lesions compared with direct clinical examination. The authors concluded that both the smartphones’ cameras and conventional macro camera had a similar performance, and that for extensive carious lesions, diagnosis based on photographic images represents a feasible option; however, the method is not accurate for detecting initial and moderate carious lesions [23].

A literature review published in 2016 by Estai et al., regarding the use of TD in detecting dental caries, concluded that the remote approach has an acceptable diagnostic value [24]. Moreover, Estai et al. investigated, in a systematic review, whether dental images were accurate for the diagnosis of dental caries and enamel defects in children and adolescents. Based on the studies included in the research, their conclusions were similar with the previously published studies; that is, for common dental diseases, photographic technology and traditional methods of dental examination yielded the least comparable results [25]. Similar results were observed by Meurer et al. (2015), who aimed to investigate whether, for the identification of common dental diseases in children and adolescents, photographic examination and subsequent image processing provided comparable accuracy to visual assessment [26].

Another review, published in 2013 by Marino and Ghanim, evaluated 59 papers published between 1992 and 2012 relevant to the teledentistry (TD) topic [27]. The authors concluded that, at that moment, TD was an area of expansion and further research was needed in optimum modalities, including costs-to-benefits ratios [14]. Similar conclusions were also drawn by Daniel and Kumar (2014), emphasizing the need for further research in the private sector [28].

One review published in 2018 investigated the benefits of teledentistry (TD) and the interest among practitioners and patients towards remote diagnosis techniques [29]. The conclusions in both papers referred to the growing interest in supporting the use of TD in different dental domains, including education. However, the authors emphasized the need for further research, with clear protocols and implementing strategies that would reduce the barriers of using TD on a large scale.

The validity of teledentistry (TD) for examination and diagnosis was investigated by Alabdullah et Daniel (2018) by performing literature research. While their findings suggested the fact that TD might be a comparable tool for face-to-face technology (regarding oral screening in schools, caries assessment, referrals, and teleconsultations), further studies are required in order to validate teledentistry as a powerful tool for dental examination and diagnosis [30].

### 4.2. The use of Teledentistry during COVID-19

During COVID-19 pandemic, and especially in lockdown periods, dentists had to overcome challenges in performing dental care. Therefore, several strategies were considered, among which teledentistry had the ability to improve access and delivery, lower the cost of oral healthcare, and eliminate disparities between the rural and urban communities [19,31].

As a result, several research works published over the last two years concentrated on the matter of optimizing dental care during the COVID-19 pandemic. The studies published, starting in 2020, were either clinical studies, surveys, or literature reviews, investigating different aspects related to the use of teledentistry.

As the COVID-19 pandemic emerged, several literature reviews investigated the use of teledentistry in different geographical regions and its application in several dental subspecialities. In this respect, some authors focused on providing an overview of the common issues in dental practice during the pandemic period along with management perspectives [32], or emphasized the possibilities of implementing telemedicine in dental practice [33,34].

In addition, for the year 2021 the interest regarding TD increased, and several articles were published. While Marya et al. [35] concentrated their research on the different modalities of using teledentistry, such as storing the data and forwarding them to a dental practitioner, monitoring patients remotely, and live video sessions between dentist and patient, as well as providing virtual oral healthcare via mobile devices, the literature review published by Kumar et al. [36] identified the three zones in which TD is used in Malaysia: educational institutions, government-based dental clinics, and private practices.

Another literature review, published by Deshpande et al., described the indications of teledentistry in general dental practice, as well as in different specializations, such as oral medicine and oral and maxillofacial surgery, orthodontics and pedodontics, prophylactic dentistry, prosthodontics, and periodontics. The authors performed an objective analysis of all the advantages of using TD in these dental domains, and they also mentioned some major pitfalls of this remote method, among which was the fact that most of the times dental treatment required a visit to a clinic; in addition, virtual examination and diagnosis were not always accurate for diagnosis [37].

Raucci-Neto et al. evaluated the level of knowledge, perceptions, and experience of teledentistry among dental practitioners in Brazil. A total of 575 respondents participated in the survey, and the results highlighted the fact that more than 80% of them had never performed TD, while a small percentage (between 2 to 5) conducted online dentist–patient interviews. Moreover, a large number of respondents considered this remote technique as being inefficient, probably due to restricted telehealth experience in the medical and dental domains [38].

Another investigation performed by Singhal et al. (2022) reviewed the use of teledentistry in Canada during the COVID-19 pandemic. The results of this environmental survey suggested that, across Canada, all of the organizations supported the use of TD during the pandemic. The authors emphasized the importance of national organizations, such as Canadian Dental Association (CDA), in recognizing the utility of teledentistry and in helping practitioners in implementing it. In addition, the authors emphasized that teledentistry could be successfully used, even in the post-pandemic era, in order to improve access and delivery of oral health with lower costs, eliminating the disparities related to oral health between rural and urban areas; being used additionally to routine visits, improving inter-professional communications, and also being environment friendly [39].

#### 4.2.1. The Use of Teledentistry in Diagnosis and Patient Monitoring

Teleconsultation allows doctors from different specialties to discuss diagnosis, treatment plans, and preservation, resulting in the resolution of more clinical cases. Furthermore, telemedicine and teledentistry have the advantages of reducing health inequalities, promoting better access to a specialized opinion, reducing waiting lists, and optimizing time and service quality [40,41]. In this context, Flores et al. (2020) investigated the acceptance of telemedicine in oral medicine. They concluded that there were similarities between in-person and remote diagnosis (by TD), with good acceptance from both patients and practitioners [40]. Similar results were obtained by da Silva et al. (2021), when investigating whether there were certain benefits from using TD in the pandemic period, in cases of patients undergoing treatment for oral head and neck cancer [41].

A retrospective study performed in Jordan, by Dar-Odeh et al., aimed to analyze dentists’ inquiries into oral infections and antimicrobial prescribing using professional groups during the lockdown. Data analysis revealed that most of the dentists’ queries via teledentistry were related to oral infections, and antibiotic or antiviral prescribing. Moreover, the authors emphasized the fact that this trend might very well continue even in the post-pandemic period [42]. A case report published by Muniz et al., in 2021, presented a 72-year-old woman with oral mucosa chemical burn, localized on the dorsal tongue surface, due to the daily usage of raw garlic. In this situation, TD proved to be an efficient tool, due to the rapidity of the patient’s professional evaluation, as well as follow-up [43].

Another pilot study published by Guidice et al., in 2020, aimed to explore whether TD was an important tool for the follow-up and management of patients who had undergone urgent surgical treatments. For the 57 patients included in the study, an evaluation of the adherence to the protocol was performed using an electronic monitoring method. For this reason, in the post-intervention, subjects had to use their smartphone’s camera to capture only the following images: (1) a picture of the surgical site; (2) a picture of the face; (3) a picture of the maximum buccal opening (including a visible ruler in the capture) after surgical treatment of the hard tissues [44]. The authors concluded that the patients’ compliance increased and that the doctor–patient relationship became stronger due to the awareness of being constantly monitored and because of the sensation of personally participating in the healing process.

Viswanathan et al. (2021) investigated the effectiveness of teledentistry in the case of a pediatric population (*n* = 208) diagnosed with cleft lip and palate, where it was known that these patients were at higher risk of developing dental caries. The pediatric team involved in the study was able to provide, via telecommunication, professional advice, reassurance, and even discharge for 11% of patients from the service of their local dental practitioners [45]. The authors emphasized the advantages of using teledentistry for this pediatric population regarding follow-up and preventive measures.

#### 4.2.2. The Use of Teledentistry for Geriatric Populations

The access to oral health care among elderly population was also investigated in the context of the COVID-19 pandemic. The sanitary crisis, in addition to certain inequalities among different social, economic, or ethnic groups, affected the access to dental services. Therefore, in a systematic review published in 2020, Aquilanti et al. investigated the feasibility of teledentistry in communities or domiciliary settings dedicated to elderly people. Based on their research, the authors concluded that TD might be a viable solution for oral care in the case of elderly populations, as this technique seemed to be as accurate as traditional face-to-face examination; cost-effective; and easily accepted by patients, caregivers, and patient’s families [46].

Two scoping reviews published in 2021 aimed to identify the applicability of TD for patients older than 60 years. Ben-Omran et al. concluded that there was evidence in favor of using teledentistry in several oral health care services, also emphasizing the need for further research [47]. Similar results were obtained by Hui Xuan Tan et al. [48], who identified the most common barriers to widely using TD, such as technical issues, lack of funding, and cognitive impairments.

Aldhuwayhi et al. (2021) used an interesting approach regarding remote dental management of a geriatric population. They performed a minireview aiming at discussing the common prosthodontic emergencies and providing recommendations for this social category. The authors concluded that, during the pandemic period, TD might be considered in case of all prosthodontic situations, emergencies, or non-emergencies, followed by portable/mobile dentistry; however, the use of personal protection equipment (PPE) in order to avoid any potential transmission if COVID-19 should be mandatory [49].

#### 4.2.3. The Use of Teledentistry in Pediatric Dentistry

In a retrospective study published by Yang et al., in 2021, the authors aimed to analyze the information regarding children’s dental teleconsultations in China. The authors performed a report including the characteristics of online consultations with pediatric patients of different ages, also reporting some of the strategies for distinguishing between dental emergencies and non-emergencies. In the pandemic period, teledentistry played an important role in patient triage, reducing the risk of transmission of highly contagious diseases such as the SARS-CoV-2 virus [50].

Sharma et al. (2021) investigated the use of teledentistry in a literature review. The authors identified the literature published before and after the COVID-19 pandemic and concluded that TD was a useful tool for providing long-term healthcare for pediatric patients, addressing the inequities related to the access of special care [51].

In a study published by Wallace et al. (2021), TD was evaluated in the context of pediatric dentistry within the Newcastle Dental Hospital, over a one-month period. The conclusions showed the potential applications of teledentistry in pediatric dentistry, following a well-established protocol. The authors highlighted the limitations of using TD due to medico-legal issues, financial implications, and data protection [52]. Similar conclusions were emphasized by Brecher et al. in research based on the implementation of teledentistry in a private pediatric dental practice located in the state of North Carolina, USA [53].

#### 4.2.4. The Use of Teledentistry in Orthodontics

By sharing orthodontics-based patient records across oral healthcare practitioners, teleorthodontics enhances treatment planning and monitoring; however, direct patient supervision and routine follow-ups throughout orthodontic therapy are still important in the orthodontics domain. Those were some of the conclusions drawn by Squires et al. (2020) [54]. Another literature review, published by Maspero et al. (2020), investigated the efficacy of teleassistance in orthodontics, as a way to manage patients at distance, in the context of the COVID-19 pandemic. They concluded that teleorthodontics could be successfully used in the context of the COVID-19 pandemic and beyond, as a result of the increasing number of technological innovations in dentistry and orthodontics. With the help of teleassistance in orthodontics, practitioners can maintain regular monitoring, manage some emergencies, or even remove dentofacial orthopedic appliances. Therefore, the authors considered teleorthodontics as having an endless potential; while, also emphasizing the fact that the data were limited and that new studies are required to evaluate the efficacy, effectiveness, and long-term results of using teledentistry in orthodontics [55].

Two case-control studies published in 2021 investigated the use of teledentistry as a monitoring tool for patients undergoing orthodontic treatment. Borujeni et al. [56] investigated the effect of teleassistance as an educational tool on the oral health status of patients undergoing fixed orthodontic treatment in the first three follow-up visits. Performed on 60 subjects, the study aimed to evaluate the effect of teledentistry in educating patients (undergoing orthodontic treatment), in order to decrease plaque accumulation. The authors concluded that, although their study had limitations, TD could be considered a useful tool for improving patient’s cooperation, due to the possibility of watching educational videos at home more than once. Similar results were obtained by Sangalli et al. [57] in their study performed on 30 subjects scheduled to start an orthodontic treatment. Compared to controls, the subjects in study group received a dental monitoring system for home use and were instructed to take monthly intra-oral scans. The results showed a significant improvement in plaque control for the study group subjects compared to controls. Therefore, the authors concluded that integrating remote monitoring systems might be an effective tool for improving plaque control and reducing carious lesions.

The same dental monitoring systems (ScanBox^®^ and cheek retractor) was used by Caruso et al. in a case report article published in 2021 [58]. The authors concluded that dental monitoring systems might be a useful tool for improving the interaction between doctor and patient, showing a significant increase in treatment efficiency. However, the authors also emphasized the fact that patients receiving such devices would have to show optimal compliance and the ability for proper use.

### 4.3. Overall Perception Regarding the Use of Teledentistry and Teleassistance during COVID-19

Dentists’ and patients’ perception of using teleassistance in dentistry was a topic of interest, especially after the emergence of the pandemic. In this context, Menhadji et al. (2021) investigated both dentists’ and patients’ attitudes towards teleassistance, to identify potential ways to improve the experience. A statistical analysis of the results revealed that the majority of the respondents had positive reactions towards teledentistry. Moreover, dentists felt competent and confident regarding online consultations [59]. Byrne and Watkinson [60] found similar results after distributing a satisfaction questionnaire to both clinicians and patients. They concluded that patients tended to prefer remote consultations, when appropriate, to those face-to-face.

A survey performed on 5370 dental practitioners in Colombia by Plaza-Ruiz et al. revealed that dentists’ knowledge, practices, and expectations increased substantially from before the COVID-19 pandemic [61]. Nevertheless, the authors emphasized existing barriers in using TD, such as the low technical skills of older dentists, insufficient financial reimbursement, and the inequalities in remote regions. Similar conclusions were also drawn by Abbas et al. [62], and by Subhan et al. [63] related to the increased interest among dental practitioners regarding teleassistance.

Patients’ opinions about teledentistry were also investigated in several research works. Three digital surveillance studies evaluated patients’ concerns regarding oral health during the pandemic. The researchers identified the queries for dental-related terms and the future trends as the COVID-19 pandemic emerged. It seemed that patients changed significantly their methods of online information searching, and that teledentistry gained popularity during the lockdown periods [64,65,66].

From the patients’ perspective, it seemed that teledentistry was considered a positive experience [67,68,69]. Factors such as convenience and communication contributed to the overall satisfaction [70].

George et al. [71] aimed to assess orthodontists’ knowledge, awareness, and attitudes regarding the use of TD during the pandemic in Kerala. Therefore, a questionnaire was distributed online, and the answers of 150 practitioners were statistically analyzed. The authors concluded that, overall, TD was acceptable. While the results did not reveal a gender difference in knowledge, attitudes, and behaviors regarding TD, younger practitioners (25–35 years old) had a more positive attitude towards teleassistance in orthodontics.

### 4.4. Future Perspectives Regarding Teleassistance in Dentistry

Mobile-health (m-health) is considered by the World Health Organization as medicine supported by mobile devices (mobile phone, patient monitoring devices, wireless devices, etc.), and over the last decade it has been applied in various medical fields [72]. A systematic review published in 2021 by Fernandez et al. aimed to determine whether teledentistry had any effects on oral health prevention in patients of all ages. Their conclusions were that teleassistance had a certain potential for becoming a useful tool in preventing and promoting oral health [73].

As the COVID-19 pandemic emerged, several Android/iOS applications were developed, in order to ease the process of online/remote consultations. Fazio et al. designed an app called LinguAPP^®^, addressed to both patients and practitioners, with the purpose of easing the remote diagnosis of oral lesions. At the moment when the study was published, the app was still used by a population from Italy [74].

As the WHO acknowledges that digital technologies are now an important part of daily life, and with the global population seeming to now be highly connected, most certainly, the rate of innovation in telemedicine is increasing as well. While the potential of digital health is still developing, there is great scope in using digital health solutions in most health domains [75].

There is a clear need to define a consistent assessment framework for teleassistance in medicine and dentistry; the development of mobile health technologies will provide value to patients and to the health-care system. However, completing a set of methodologies and approaches to properly evaluate the new mHealth solutions (from patients, to medical practitioners to health care authorities) will facilitate rational decisions in the choice and use of digital solutions.

Nowadays, although the literature is developing regarding subjects covering a large area related to dentist’s expectations and attitude towards teledentistry, special attention needs to be addressed towards understanding and developing this subject. Teledentistry is expanding its use, from pediatric dentistry, to odontology, periodontology, prosthodontics, oral surgery, and orthodontics. There are a large number of studies, which show a trend towards implementing new and technological digitalization in medicine and dentistry.

We included four articles discussing the topic of pediatric dentistry, a six publications concerning orthodontics, four publications regarding geriatric dentistry, and nine publications investigating the use of TD for the diagnosis and monitoring of patients.

There is still a need to introduce guidelines in teledentistry, starting with examination procedures, home care strategies, and treatment evolution monitoring. Nevertheless, teledentistry cannot completely substitute classic dentistry, due to the fact that it is a practical specialty. However, artificial intelligence is expanding; therefore, TD could be integrated into those platforms.

We believe that the use of TD will continue to rise, even in the post-pandemic period. Using TD is a safe and reliable method and can address various healthcare settings and pandemic situations, as well for healthy individuals (adults and children) and for people with special needs. TD represents a challenge for practitioners and researchers; nevertheless, there are still many problems that need to be identified, from ethical concerns to individual privacy and personnel training.

### 4.5. Limitations of Our Study

This study’s limitations concern, first of all, the inclusion of a limited number of articles compared to the available publications on telemedicine and teledentistry. However, we decided to include only articles that were relevant to the aims of our research. Our aim was an integrative approach regarding the practitioner’s and patient’s perspective toward easing the diagnostic tools and treatment outcomes by means of teledentistry.

Secondly, designing this review as an integrative review, rather than a systematic review was due to the high heterogeneity of data included, and the scarcity of available data on different aspects regarding teledentistry.

In addition, we also want to emphasize the fact that while teledentistry became increasingly popular as the COVID-19 pandemic emerged, strict protocols about its use and evaluation of the clinical outcomes, long-term use, and economic analysis are required. In addition, aspects such as backup communication systems, appropriate internet sources, technical support, and variations in the knowledge and skills of dental practitioners are expected to limit the use of teledentistry on a large scale, at least in the near future.

## 5. Conclusions

Considering the results of this integrative review, as well as the limitations of our research, there is clear evidence that the interest in teledentistry and teleassistance in the dental domain increased rapidly, especially in the context of the COVID-19 pandemic. Compared to the pre-pandemic period, the number of studies regarding teledentistry and teleassistance increased substantially, as well as patients’ interest in this topic.

Teledentistry can be successfully used in general dentistry, pediatric dentistry, and orthodontics, as well as geriatric dentistry, being a helpful tool for the management of preliminary emergencies (e.g., prescribing suitable antibiotic therapies), aiding in specialist consultations (facilitating treatment planning), or in avoiding follow-up visits (e.g., after performing emergency procedures). However, when using teledentistry, some crucial steps in the diagnosis process cannot be performed (such as palpation and percussion), as well as clinical procedures (restoration, endodontic treatments, extractions), and these require clinic visits.

As the trend is to continue further using different digital technologies, methodologies and specific protocols should be developed. There is a great need for developing guidelines, from the regional regulatory authorities along with the World Health Organization (WHO), in order to regulate the proper use of all the methods and technologies used in digital health and teleassistance.

Therefore, while dental practitioners should be encouraged to keep updated on the new technologies, patients should also be constantly informed about their options in receiving special oral health care. These new modalities need to be considered by governments and other health care authorities, to ensure a proper structure and in order to obtain the best long-term results.

## Figures and Tables

**Table 1:** Inclusion and exclusion criteria.

Criterion	Inclusion	Exclusion
Time period	Publications available between January 2012 and January 2022	All publications published before January 2011
Language	English	Non-English
Type of articles	Clinical trials Randomized controlled trials Surveys Observational studies Reviews and systematic reviews	Editorials Short communications Letters to editor No full-text available Publications outside the area of investigation
